# The effect of almonds on vitamin E status and cardiovascular risk factors in Korean adults: a randomized clinical trial

**DOI:** 10.1007/s00394-017-1480-5

**Published:** 2017-07-10

**Authors:** Hana Jung, C.-Y. Oliver Chen, Jeffrey B. Blumberg, Ho-Kyung Kwak

**Affiliations:** 1 Department of Human Ecology, Korea National Open University, 86, Daehak-ro, Jongno-gu, Seoul, 03087 Republic of Korea; 2 Antioxidants Research Laboratory, Jean Mayer USDA Human Nutrition Research Center on Aging, Tufts University, Boston, MA 02111 USA

**Keywords:** Almonds, Cholesterol, α-Tocopherol, Cardiovascular risk factor, Oxidative stress, Inflammation, Human

## Abstract

**Purpose:**

Almonds have shown to beneficially modify some cardiovascular risk factors in clinical trials conducted in diverse ethnic populations but this relationship has never been tested in Koreans. Thus, we tested the impact of almonds consumed as a snack within the context of a typical Korean diet on cardiovascular risk factors.

**Methods:**

We conducted a randomized, crossover trial in a free-living setting with a 2-week run-in period, two 4-week intervention phases, and a 2-week washout period between interventions. Eighty four overweight/obese participants (11 M/73 F; 52.4 ± 0.6 year; 25.4 ± 0.22 kg/m^2^) consumed either 56 g of almonds or isocaloric cookies daily for 4 weeks.

**Results:**

Mean % daily energy intake at baseline was 64.8, 21.3, and 14.9% from carbohydrate, fat, and protein, respectively. The addition of 56 g of almonds daily decreased carbohydrate energy to 55.0%, increased fat to 32.0%, and maintained protein at 14.7%. Consuming the almonds increased intake of MUFA by 192.3%, PUFA by 84.5%, vitamin E by 102.7%, and dietary fiber by 11.8% and decreased % energy from carbohydrate by 14.1%. Total caloric intake was increased by the almonds, but body weight, waist circumference, and body composition were not affected. Almonds in overweight and obese Korean adults decreased TC, LDL-C, and non-HDL-C by 5.5, 4.6, and 6.4%, respectively, compared to the cookie control (*P* ≤ 0.05). Almonds increased plasma α-tocopherol by 8.5% (*P* ≤ 0.05) from the baseline and tended to increase its value as compared to cookies (*P* = 0.055). Neither the almonds nor cookies altered plasma protein carbonyls, MDA or oxLDL. Of serum inflammatory markers, IL-10 was decreased by almond intake (*P* ≤ 0.05), and ICAM-1, IL-1β, and IL-6 tended to be lower with almonds, compared to the cookies.

**Conclusions:**

Almonds at 56 g/day consumed as a snack favorably modified the Korean diet by increasing MUFA, PUFA, vitamin E, and dietary fiber intake and decreasing % energy intake from carbohydrate. Almonds also enhanced plasma α-tocopherol status and serum TC and LDL-C in overweight and obese Koreans. Thus, including almonds in typical Korean diets as a snack can help healthy overweight/obese individuals improve nutritional status and reduce their risk for CVD.

**Electronic supplementary material:**

The online version of this article (doi:10.1007/s00394-017-1480-5) contains supplementary material, which is available to authorized users.

## Introduction

Cardiovascular disease (CVD) is the leading cause of mortality and morbidity in the world [1], including in South Korea [2]. Risk factors for CVD are numerous and include dyslipidemia, hypertension, smoking, obesity, sedentary lifestyle, stress, family history of CVD, and insulin resistance [1, 3]. The prevalence of hypercholesterolemia (TC ≥240 mg/dL) among adults ≥30 year in South Korea was 14.6% according to the data of the 2014 Korea National Health and Nutrition Examination Survey (KNHANES) [2], which is comparable to values reported in America (15%, ≥20 year) [4] and China (14.7%, ≥18 year) [5]. Importantly, the prevalence of hypercholesterolemia has been gradually increasing during the last decade in South Korea [2]. Overweight/obesity and diet are two principal modifiable factors affecting the development of hypercholesterolemia [6]. In South Korea, the incidence of obesity among adults (BMI ≥25, based on the WHO obesity guideline for the Asia–Pacific region) [7] is 37.7% in men and 25.3% in women [2]. To reduce the risk of CVD, approaches to mitigating modifiable risk factors, such as unhealthy diets and sedentary lifestyles, are recommended to be undertaken by all people, especially those at increased risk associated with unmodifiable factors such as age and genetics.

The traditional Korean diet is rich in carbohydrates and low in fats, with carbohydrate (CHO), fat, and protein contributing to 72.1, 13.4, and 14.5%, respectively, of total energy intake [8]. Further, in the KNHANES data [2], the contribution of CHO to total energy intake is 63.8%, which is even higher in adults ≥50 year. In the typical Korean diet, grains and grain-based foods contribute about 48%, and white rice contributes about 25% of energy [2]. This rice-based dietary pattern predisposes Koreans to obesity, dyslipidemia and diabetes [9]. Besides being a CHO-predominate dietary pattern, the typical Korean diet is inadequate in some micronutrients. For example, 70% of Korean adults do not consume the estimated average requirement for calcium recommended by the Dietary Reference Intakes for Koreans (KDRI) [2]. Korean adults consume over 50% of vitamin E in γ-tocopherol form and only 22% in α-tocopherol form [10]. Furthermore, 23% of Korean adults have plasma α-tocopherol concentration lower than 12 μmol/L, a threshold level of vitamin E deficiency, and 90% of Korean adults have its level below 20 μmol/L [10], which is associated with increased CVD risk [11, 12]. Both α- and γ-tocopherol have been reported to exert anti-inflammatory actions [11]. Thus, incorporating foods high in quality fats and micronutrients into the Korean diet would be expected to improve overall nutrition status.

Nuts, including tree nuts and peanuts, contain a wide range of beneficial nutrients, such as fiber, protein, unsaturated fats, vitamins, minerals, and phytosterols and other phytochemicals [13, 14]. Of all tree nuts, almonds have been frequently demonstrated in clinical studies to lower blood glucose and cholesterol and attenuate biomarkers of inflammation and oxidative stress, all are risk factors of CVD [15–21]. These benefits are mainly ascribed to their nutrient composition being low in saturated fatty acids (SFA) and rich in unsaturated fatty acids (91–94% fats are oleic acid and linoleic acid) and α-tocopherol and containing fiber, phytosterols, and proteins [20]. Thus, incorporating almonds into typical Korean diets might be expected to support cardiovascular health and improve the status of certain nutrients, such as vitamin E.

The benefit to cardiovascular health of almond consumption has been demonstrated in people living in Canada, China, Taiwan, United States, and United Kingdom, but not in South Korea. However, the health benefit of any specific food is subject to the influence of many physiological, genetic, dietary, and environmental factors. As ethnicity and background diet may modulate the bioefficacy of almond nutrients, we tested the impact of almonds on CVD risk factors in overweight/obese Korean adults. We hypothesized that almonds could improve vitamin E status and CVD risk factors including lipid profile, oxidative stress, and inflammation.

## Subjects and methods

### Subjects

**Fig. 1:**
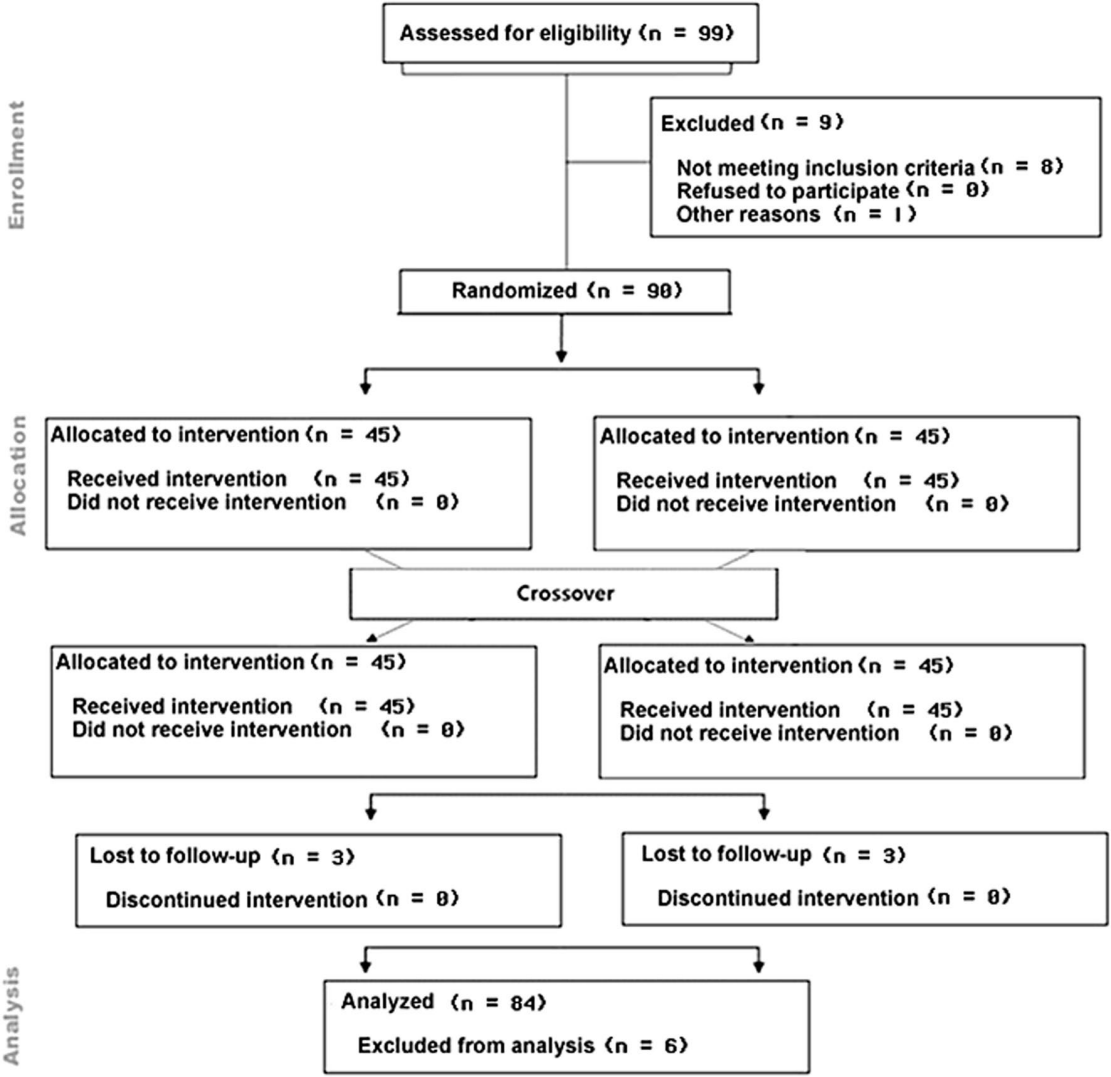
CONSORT of flow chart of study

Subjects were recruited in the Seoul metropolitan area by advertising on the website of Korea National Open University and the campus bulletin boards (Fig. 1). The eligibility criteria included: (1) men and women aged 45–69 year, (2) BMI 23–29.9 kg/m^2^ or waist circumference ≥85 cm for women, ≥90 cm for men, (3) free of any diagnosed chronic disorders or acute inflammatory diseases 2 year prior to the enrollment, (4) no known allergies to nuts, (5) nonsmoking (or ceased smoking for ≥1 year), (6) not taking any vitamin supplements, functional foods or hormone replacement therapy for the last 1 month prior to the enrollment, (7) not taking any medications known to affect lipid metabolism, such as statins, (8) consuming typical Korean diet. Subjects who had passed an initial phone screening were invited for on-site screening to confirm their eligibility. The study was approved by the Institute Review Board of the Korea National Open University, and informed consent was obtained from each participant before the conduct of any study element was performed. The trial was registered in the Institute Review Board of the Korea National Open University: KNOU IACF (ABN01-201412-11-28).

### Experimental design

**Fig. 2:**
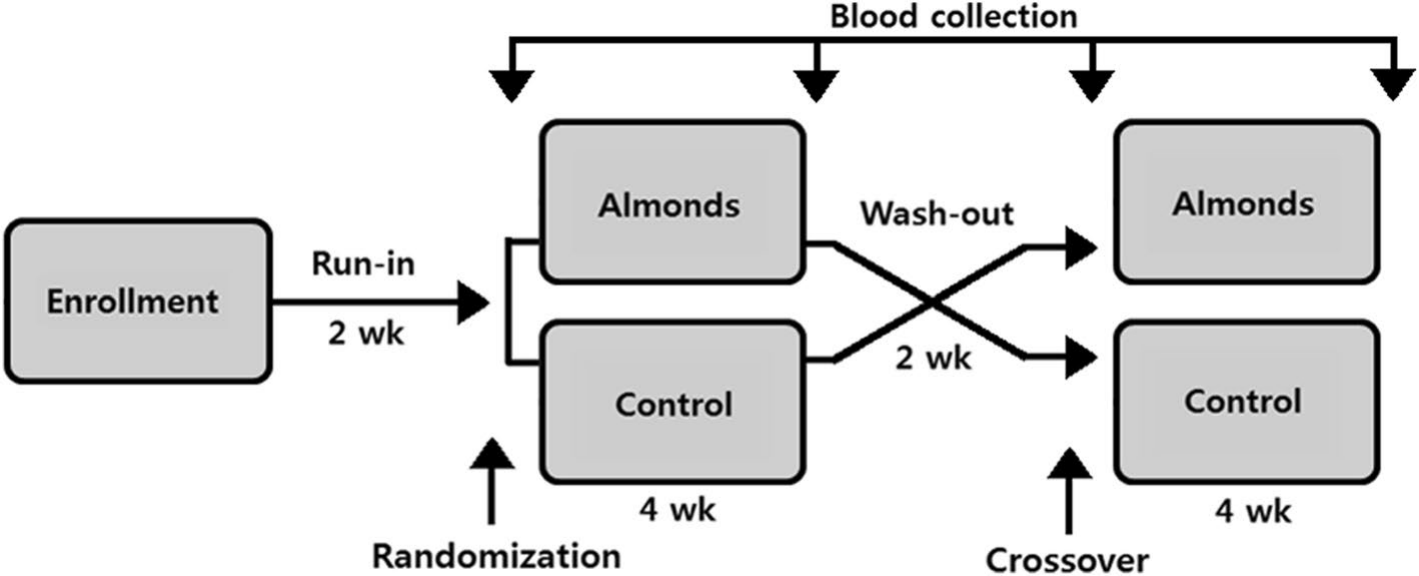
Study design

The study was a 12-week randomized crossover intervention trial in a free-living setting (Fig. 2). During the study, subjects were asked to consume their habitual diet without nuts. After 2 weeks of run-in period, eligible subjects were randomly assigned to one of two sequences [almond then control (AC) and control then almond (CA)] by asking them to draw a card (blue card: AC; orange card: CA). Subjects were then instructed to consume either 56 g/day of roasted almonds or 70 g/day of isocaloric home-made cookies as a snack for 4 weeks in the first intervention phase. After the 2-week washout period, subjects consumed the alternate food for another 4 weeks in the second phase. The daily dose of almonds and cookies was individually packaged. Almonds or cookies were provided to subjects every 2 weeks. Subject compliance was assessed with a diary calendar and counting returned packages. Four 3-day dietary records (2 weekdays and 1 weekend day) were collected in the final week of the run-in, washout, and intervention phases. Nutrient intakes were assessed using a computer aided nutritional analysis program for professionals (CAN pro 4.0, The Korean Nutrition Society).

### Study foods

Roasted almonds were generously provided by the Almond Board of California. The cookies were made with white flour, butter, sugar, egg, baking powder, and salt in a local bakery. After preparation, the cookies were stored in sealed aluminum foil zipper bag pouches, at room temperature, protected from light and air. The nutrient composition of almonds and cookies is presented in Table 1.

**Table 1:** Nutrient composition of almonds and cookies

Nutrients		Cookies per 70 g	Roasted almonds per 56 g
Calories	kcal	340	340
Moisture	g	1.5	1.4
Protein (% kcal)	g	4 (4.7)	12 (14.1)
CHO (% kcal)	g	47.3 (55.6)	5.5 (6.5)
Total sugar (% kcal)	g	16.0 (18.8)	2.8 (3.3)
Total fat (% kcal)	g	15 (39.7)	30 (79.4)
SFA (% kcal)	g	8.4 (22.2)	2.3 (6.1)
MUFA (% kcal)	g	3.2 (8.5)	19 (50.3)
PUFA (% kcal)	g	0.6 (1.6)	7.3 (19.3)
Total dietary fiber	g	0.7	6.2
Calcium	mg	14	150
Iron	mg	0.4	2.1
Magnesium	mg	9	160
Phosphorus	mg	67	270
Potassium	mg	58	400
Sodium	mg	152	2
Zinc	mg	0.4	1.9
α-tocopherol	mg	0.41	14.0

*CHO* carbohydrate, *SFA* saturated fatty acids, *MUFA* monounsaturated fatty acids, *PUFA* polyunsaturated fatty acids

### Anthropometric analyses

Body weight, height, body composition, and blood pressure were measured before and after each intervention phase. Body weight and height were measured using an anthropometer (HC-1000, Cas Korea, Seoul, Korea). BMI was calculated using the weight/height^2^ (kg/m^2^) formula. Waist and hip circumferences were measured using a measuring tape. Body composition was assessed using bioelectrical impedance analysis (InBody720, Bio-space, Seoul, Korea). After ≥10 min of rest, blood pressure was determined using an automatic blood pressure monitor (TM-2655P, Bio-space).

### Blood collection

After a 12-h fast, venous blood was collected from the brachial vein of one arm before and after each intervention phase. Whole blood was centrifuged at 1500×*g* for 10 min at 4 °C. Plasma and serum were separated and aliquots were stored in −80 °C until biochemical analyses were performed.

### Biochemical analyses

Serum triglyceride (TG), total choesterol (TC), low-density lipoprotein cholesterol (LDL-C), high-density lipoprotein choesterol (HDL-C), glucose, high-sensitive C-reactive protein (hs-CRP), apolipoprotein A (apo A), and apolipoprotein B (apo B) were analyzed using an automated biochemical analyzer (Selectra E, Vital Scientific N.V., Dieren, The Netherlands). The intra- and inter-day coefficients of variation (CV) were 1.9 and 5.3% for TG, 0.5 and 8.4% for TC, 3.1 and 4.4% for LDL-C, 3.8 and 7.5% for HDL-C, 1.3 and 9.6% for glucose, 7.7 and 8.4% for hs-CRP, 1.6 and 12.6% for apo A, and 8.8 and 5.4% for apo B, respectively. Plasma protein carbonyls and oxidized low-density lipoprotein cholesterol (oxLDL) were measured using commercial kits (Cell Biolab, San Diego, CA, USA) and a microplate reader (Fluostar Optima, BMG Labtech, Durham, NC, USA). The intra- and inter-day CV for protein carbonyls were 6.8 and 12.2% and for oxLDL were 2.3 and 9.8%, respectively. Tumor necrosis factor-α (TNF-α), interleukin (IL)-1β, IL-6, and IL-10 in serum were determined using a cytometric cytokine bead array human enhanced sensitivity master buffer kit (BD Biosciences, San Jose, CA, USA) and an Accuri C6 flow cytometer (BD Biosciences). Soluble vascular cell adhesion molecule-1 (VCAM-1) and intercellular adhesion molecule-1 (ICAM-1) were measured using commercial kits (R&D Systems, Abingdon, UK). The intra- and inter-day CV for VCAM-1 were 1.3 and 12.8% and for ICAM-1 were 5.2 and 11.0%, respectively. Plasma α- and γ-tocopherol were determined using a high-performance liquid chromatography (HPLC) with photo-diode array (PDA) method according to Liu et al. [22]. The intra- and inter-day CV for α-tocopherol were 1.8 and 4.4% and for γ-tocopherol were 1.5 and 5.2%, respectively. Plasma malondialdehyde (MDA) was determined by a reverse-phase HPLC method with the thiobarbituric acid–MDA conjugate being injected onto a C18 column and then quantified with fluorescence detection (Ex515/Em553 nm) according to Volpi and Tarugi [23]. The intra- and inter-day CV for MDA were 3.3 and 7.3%, respectively.

### Statistical analyses

The results are expressed as mean ± standard deviation (SD). The data were analyzed using SAS 9.4 for Windows (SAS Institute Inc, Cary, NC). Student’s *t* test was performed to compare nutrient intakes between the time points. Student’s *t* test was also used to assess the significance of differences in biochemical biomarkers between cookie baseline and almond baseline, as well as between before and after each intervention phase. The mixed model was performed (SAS: Proc Mixed) to assess the treatment effect to control variance in the outcome by accounting for the crossover nature of the study. In this model, subject was designated as the random effect and treatment and sequence were designated as the fixed effects. Significance was considered at *P* ≤ 0.05.

## Results

### Subject characteristics

One hundred forty five people were phone screened, and 99 of them were invited to on-site screening to assess eligibility. Ninety eligible participants were enrolled, and 84 [11 M and 73 W, aged 45–62 year (52.4 ± 0.6 year)] completed the study with full compliance (Fig. 1). Five participants (5 F/1 M) dropped out from the trial due to time commitment (*n* = 3) and abdominal discomfort caused by almonds in the first week (*n* = 2). In addition, the data from one participant were not included in data analysis because of the elevated inflammatory status (hs-CPR and IL-6) that was three times of the SD. The percent of overweight (23≤ BMI <25) and obese (BMI ≥25) subjects was 39.3 and 51.2%, respectively (WHO obesity guideline for Asia–Pacific region) [7], and central adiposity (men, waist circumference ≥90, women, waist circumference ≥85) [7]  was 96.4%. The proportion of participants with normal levels of TG [≤1.7 mmol/L (≤150 mg/dL)], TC [≤5.2 mmol/L (≤200 mg/dL)] and LDL-C [≤3.4 mmol/L (≤130 mg/dL)] was 91.7, 57.1, and 57.1%, respectively, and 76.2% of the participants had normal HDL-C [>1.0 mmol/L (>40 mg/dL)] [3]. Most participants (97.6%) had a normal fasting glucose value [≤5.6 mmol/L (≤100 mg/dL)]. Plasma α-tocopherol ranged from 20.7 to 50.0 μmol/L. The proportion of participants with intermediate risk and high risk levels of hs-CRP (>1.0, AHA/CDC guideline) [24] was 19.0%.

### Dietary intake

Compared with the values from the pre-almond phase (wk 0), almonds increased total energy by 12.3%, MUFA by 192.3%, PUFA by 84.5%, dietary fiber by 11.8%, vitamin E by 102.7%, calcium by 19.7%, and magnesium by 190.8% and decreased % energy from CHO by 14.1% (Table 2). As compared to the week 4 value of the cookie phase, mean % daily energy intake from CHO was 5.7% lower during the almond phase (*P* ≤ 0.001), as well as 5.8% higher from fats (*P* ≤ 0.001). The addition of almonds also significantly increased the intake of PUFA and MUFA, dietary fiber, and vitamin E by 67.9, 107.8, 22.5, and 114.4%, respectively, as compared to the cookie control. Intake of energy, calcium, and magnesium were significantly increased by 6.2, 20.6, and 185.0%, and SFA and cholesterol were significantly decreased by 44.0 and 14.1% during the almond phase compared to the cookie phase.

**Table 2:** Dietary intake data of the subjects before and after each phase^a^

	Cookie		Almond		*P* value^b^
	Week 0	Week 4	Week 0	Week 4	
Energy (kcal)	1678.1 ± 374.3	1803.3 ± 332.3	1704.3 ± 343.4	1914.4 ± 340.6^†^	0.0224
Carbohydrate (g) (% of energy)	264.3 ± 62.9 (63.1 ± 6.4)	275.3 ± 60.2 (60.7 ± 5.9)	273.1 ± 4.18 (64.0 ± 7.0)	264.4 ± 58.5 (55.0 ± 6.1^‡^)	≤0.001^c^
Protein (g) (% of energy)	63.6 ± 16.3 (15.2 ± 2.4)	61.1 ± 14.0 (13.5 ± 1.9)	63.7 ± 15.0 (14.9 ± 2.2)	70.5 ± 15.9 (14.7 ± 2.3^‡^)	≤0.001^c^
Fat (g) (% of energy)	42.4 ± 14.8 (22.5 ± 5.2)	52.4 ± 11.4 (26.2 ± 4.3)	42.1 ± 5.0 (22.0 ± 4.5)	67.9 ± 14.0 (32.0 ± 4.5^‡^)	≤0.001^c^
SFA (g) (% of energy)	6.3 ± 4.0 (3.4 ± 2.1)	16.1 ± 4.5 (8.2 ± 2.3)	7.1 ± 4.2 (3.8 ± 2.2)	9.0 ± 3.8^‡^ (4.3 ± 1.8)	≤0.001
MUFA (g) (% of energy)	7.9 ± 4.6 (4.2 ± 2.4)	12.8 ± 5.1 (6.5 ± 2.5)	9.1 ± 5.0 (4.8 ± 2.7)	26.6 ± 5.4^‡^ (12.8 ± 2.8)	≤0.001
PUFA (g) (% of energy)	6.7 ± 3.2 (3.5 ± 1.4)	7.8 ± 2.9 (3.9 ± 1.4)	7.1 ± 2.8 (3.8 ± 1.3)	13.1 ± 3.1^‡^ (6.3 ± 1.5)	≤0.001
Cholesterol (mg)	316.7 ± 163.6	345.8 ± 131.6	292.6 ± 158.6	297.2 ± 146.5^†^	0.0413
Fiber (g)	21.0 ± 5.0	20.9 ± 6.0	22.9 ± 6.1	25.6 ± 4.4^‡^	≤0.001
Vitamin E (mg)	14.1 ± 5.0	13.9 ± 4.0	14.7 ± 4.4	29.8 ± 4.6^‡^	≤0.001
Calcium (mg)	478.8 ± 139.9	500.8 ± 164.5	504.6 ± 178.9	603.9 ± 174.6^‡^	≤0.001
Magnesium (mg)	66.5 ± 31.6	74.7 ± 25.4	73.2 ± 28.8	212.9 ± 37.0^‡^	≤0.001

Data are expressed as mean ± SD. Nutrient intakes were evaluated using a computer aided nutritional analysis program for professionals (CAN pro 4.0, The Korean Nutrition Society)

*SFA* Saturated fatty acids, *MUFA* monounsaturated fatty acids, *PUFA* polyunsaturated fatty acids

^a^Dietary record was collected before each phase (week 0) and during the final week of each phase (week 4)

^b^Comparison of values at the end of almond and cookie phases, tested using the mixed model

^†^ *P* ≤ 0.05 and ^‡^ *P* ≤ 0.001, comparison between almond (week 4) vs. cookie (week 4), tested using Student’s t-test

### Anthropometric indices and blood pressure

At the end of the intervention, neither almonds nor cookies altered body weight, waist circumference, body composition, and blood pressure as compared to the respective baseline value (Supplemental Table 1). The overall mean body weight, waist circumference, body fat, and systolic and diastolic blood pressures were 66.2 ± 8.7, 89.5 ± 5.9, 43.1 ± 8.6, 122.1 ± 16.1, and 79.4 ± 10.2, respectively.

### Lipid profiles and fasting glucose

Mean TG and HDL-C values were not significantly changed during the intervention (Table 3). However, when compared to the corresponding pre-intervention value, almonds tended to reduce TG by 8.0% (*P* = 0.189) while the cookies had no impact. As compared to the end value of the cookie phase, almonds decreased TC, LDL-C, and non-HDL-C by 5.5, 4.6 and 6.4%, respectively (*P* ≤ 0.05), and tended to decrease serum apo B by 4.6% (*P* = 0.109). Neither almonds nor cookies affected blood glucose status.

**Table 3:** Lipid profiles and fasting glucose of the subjects before and after each phase

	Cookie		Almond		*P* value^a^
	Week 0	Week 4	Week 0	Week 4	
TG (mmol/L)	1.09 ± 0.48	1.08 ± 0.50	1.12 ± 0.49	1.03 ± 0.46	0.1583
TC (mmol/L)	5.08 ± 0.93	5.28 ± 1.01	4.99 ± 0.88	4.99 ± 0.91	0.0198
LDL-C (mmol/L)	3.29 ± 0.70	3.41 ± 0.78	3.23 ± 0.68	3.19 ± 0.70	0.0222
HDL-C (mmol/L)	1.24 ± 0.28	1.28 ± 0.25	1.21 ± 0.26	1.26 ± 0.30	0.9573
Apo A (mg/dL)	150.59 ± 24.71	157.33 ± 25.84	150.62 ± 25.84	153.95 ± 25.97	0.1319
Apo B (mg/dL)	91.09 ± 19.10	92.31 ± 20.70	89.34 ± 19.02	87.98 ± 18.83	0.1091
Apo B/Apo A	0.62 ± 0.16	0.60 ± 0.16	0.61 ± 0.15	0.58 ± 0.14	0.6557
LDL-C/HDL-C	2.81 ± 0.91	2.76 ± 0.78	2.79 ± 0.81	2.67 ± 0.80	0.2310
TC/HDL-C	4.31 ± 1.26	4.26 ± 1.03	4.29 ± 1.09	4.15 ± 1.09	0.3578
Non-HDL-C^b^ (mmol/L)	3.85 ± 0.92	3.99 ± 0.96	3.78 ± 0.85	3.74 ± 0.88	0.0121
Glucose (mmol/L)	4.80 ± 0.51	4.83 ± 0.68	4.82 ± 0.57	4.76 ± 0.55	0.1125

Data are expressed as mean ± SD

*TG* triglyceride, *TC* total cholesterol, *LDL-C* low-density lipoprotein cholesterol, *HDL-C* high-density lipoprotein cholesterol, *Apo A* apolipoprotein A, *Apo B* apolipoprotein B

^a^Comparison of values at the end of almond and cookie phases, tested using the mixed model

^b^Non-HDL-C: total cholesterol-high-density lipoprotein cholesterol

### Plasma tocopherol profiles

**Fig. 3:**
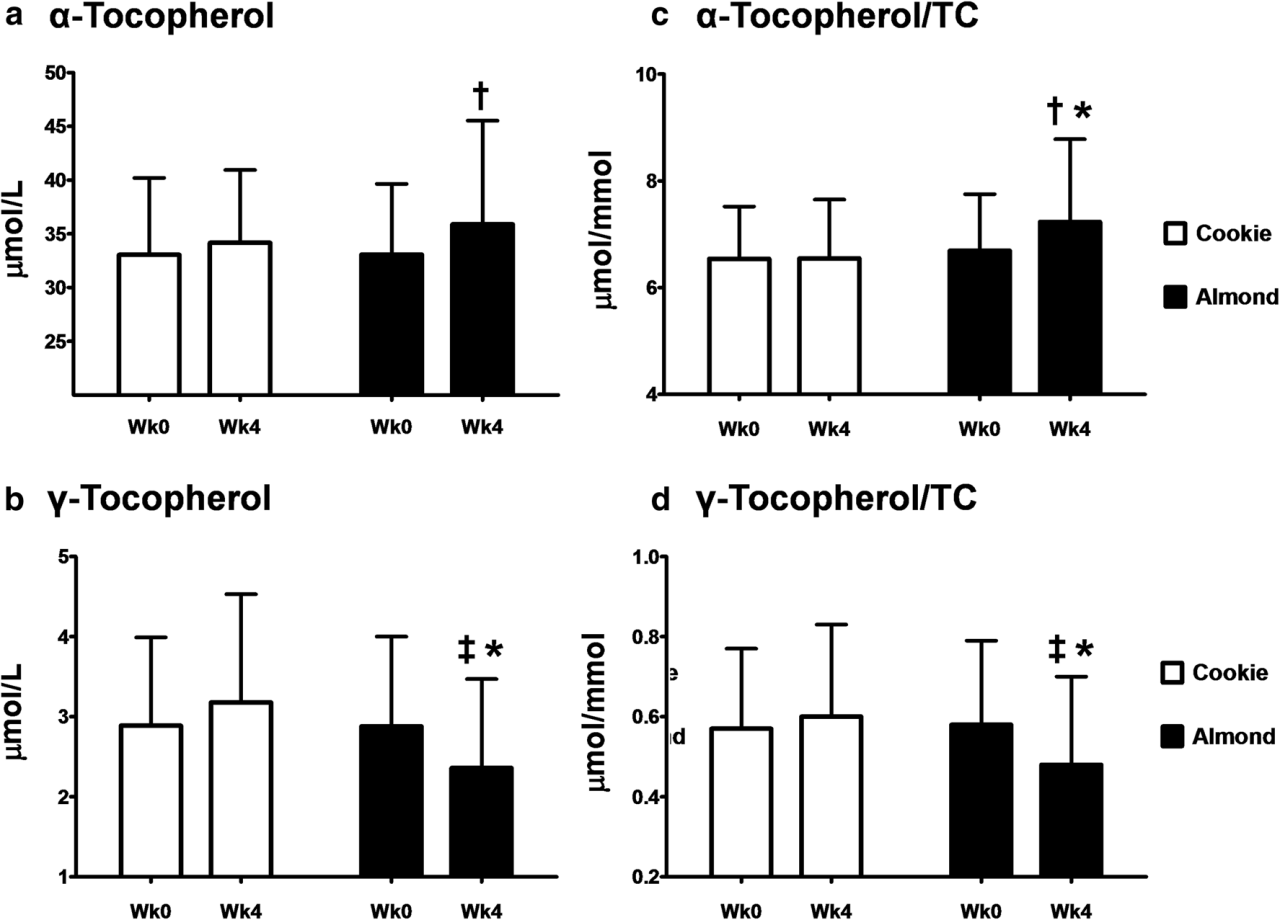
Changes in α- (**a**) and γ-tocopherol (**b**) and the ratio of tocopherol to total cholesterol (**c**, **d**) in subjects consuming either almonds or cookies for 4 weeks. Data are expressed as mean ± SD. ^†^ *P* ≤ 0.05 and ^‡^ *P* ≤ 0.01, means within each treatment differ between pre- and post-intervention values, tested by Student’s *t* test. *P ≤ 0.001, post-intervention means differ between almonds and cookies, tested using the mixed model

After 4 weeks of almond consumption, plasma α-tocopherol concentration tended to be higher (*P* = 0.055) and γ-tocopherol was lower (*P* ≤ 0.001) than cookie consumption (Fig. 3). When compared with the respective pre-intervention value, almonds increased α-tocopherol concentration by 8.5% (*P* ≤ 0.05) and decreased γ-tocopherol by 18.1% (*P* ≤ 0.01). At the end of the intervention, almonds increased the ratio of α-tocopherol to TC by 10.4% (*P* ≤ 0.001) and decreased the ratio of γ-tocopherol to TC by 20.0% (P ≤ 0.001), as compared to cookies. Compared with the pre-intervention value, almonds increased the ratio of α-tocopherol to TC by 8.0% (*P* ≤ 0.05) and decreased the ratio of γ-tocopherol to TC by 17.2% (*P* ≤ 0.01).

### Biomarkers of oxidative stress and inflammation

Neither almonds nor cookies affected the selected biomarkers of oxidative stress, i.e., plasma protein carbonyls, MDA, and serum oxLDL (Table 4). Almonds did modulate some biomarkers of inflammation as compared to cookies. Almonds decreased post-intervention serum IL-10 concentration by 22.2%, as compared to cookies (*P* ≤ 0.05). Serum ICAM-1 (*P* = 0.187), IL-1β (*P* = 0.137) and IL-6 (*P* = 0.142) tended to be lowered at the end of the almond phase than the cookie phase. Other biomarkers of inflammation, serum VCAM-1, TNF-α, and hs-CRP, showed no notable changes during the intervention.

**Table 4:** Biomarkers of oxidative stress and inflammation of the subjects before and after each phase

	Cookie		Almond		*P* value^a^
	Week 0	Week 4	Week 0	Week 4	
Oxidative stress markers					
Protein carbonyl (mmol/mL)	0.65 ± 0.41	0.69 ± 0.41	0.68 ± 0.38	0.68 ± 0.37	0.2765
Malondialdehyde (μmol/L)	1.93 ± 0.38	1.93 ± 0.40	1.94 ± 0.37	1.93 ± 0.40	0.8436
Oxidized LDL (mg/mL)	6.39 ± 3.76	5.79 ± 4.28	5.71 ± 3.67	5.00 ± 3.40	0.3330
Inflammatory markers					
ICAM-1 (ng/mL)	162.54 ± 66.01	156.30 ± 61.98	169.39 ± 67.53	156.81 ± 61.33	0.1872
VCAM-1 (ng/mL)	486.19 ± 140.53	472.94 ± 132.69	492.05 ± 142.58	474.11 ± 133.17	0.7731
IL-10 (pg/mL)	0.23 ± 0.16	0.26 ± 0.24	0.25 ± 0.27	0.20 ± 0.16	0.0393
IL-1β (pg/mL)	0.07 ± 0.08	0.07 ± 0.08	0.07 ± 0.07	0.06 ± 0.07	0.1372
IL-6 (pg/mL)	0.70 ± 0.70	0.66 ± 0.63	0.70 ± 0.67	0.57 ± 0.58	0.1419
TNF-α (pg/mL)	0.10 ± 0.09	0.10 ± 0.78	0.11 ± 0.09	0.10 ± 0.09	0.9514
hs-CRP (mg/dL)	0.11 ± 0.31	0.11 ± 0.21	0.11 ± 0.21	0.08 ± 0.11	0.3205

Data are expressed as mean ± SD

*ICAM* intercellular adhesion molecule, *VCAM* vascular cell adhesion molecule, *IL* interleukin, *TNF-α* tumor necrosis factor-α, *hs-CRP* high-sensitive C-reactive protein

^a^Comparison of values at the end of almond and cookie phases, tested using the mixed model

## Discussion

Several studies support a cardiometabolic benefit of almond consumption based on their association with improvements to lipid profile, blood glucose, inflammation, and oxidative stress [15–19]. However, such benefits are always subject to the influence of other factors, including background diet and ethnicity. As the impact of almonds has not been examined in Koreans, this study tested whether almonds consumed as a snack could improve vitamin E status, lipid profile, and biomarkers of oxidative stress and inflammation in overweight/obese Korean adults.

Almonds are a nutrient dense food because they are a particularly good source of unsaturated fatty acids, α-tocopherol, arginine, magnesium, copper, calcium, and potassium [20]. The addition of almonds to a daily diet may improve the nutritional quality of diverse populations by increasing the intake of unsaturated fatty acids, fiber, magnesium, and α-tocopherol [25, 26, 27]. We found that almonds at 56 g/day improved the nutrition quality of free-living Koreans consuming a typical national diet. The change in energy distribution from CHO to fat during the almond phase shifted the dietary pattern to one more consistent with the National Cholesterol Education Program (NCEP) guidelines for healthy American adults, i.e., 50–60% calories from CHO, 15% from protein, and 25–35% from fat (≤7% of calories from SFA, up to 10% from PUFA, and up to 20% from MUFA) [28]. Even though the percent of energy from CHO during the almond phase remained within the recommended range for Koreans (55 ~ 65%) [29], we found almonds consumed as a snack enabled a larger change in the contribution of CHO and fat to total energy as compared to the studies with participants consuming Western diets [25, 26]. Almonds increased the intake of vitamin E, fiber, MUFA, and PUFA. Particularly, they doubled vitamin E intake from 14.7 to 29.8 mg/day and elevated fiber intake up to the level (≥25 g/day) recommended in the Korean Guidelines for the Management of Dyslipidemia [3]. Furthermore, almonds enhanced calcium and magnesium intakes closer to the recommended levels for Korean adults ≥45 year (700 ~ 800 mg for calcium and 280 ~ 370 mg for magnesium) [29].

The addition of almonds (56 g/day) as a snack increased mean daily energy intake by 12%, but did not alter body weight. Despite the provision of 15 g fat and 169 kcal energy from a 28 g of serving, several clinical trials reveal that almonds show a beneficial or null effect on body weight [26, 30–33], likely due to a combination of food displacement and incomplete calorie absorption. For example, Novotny et al. [34] reported that 32% of calories in almonds was not absorbed and excreted in stool as compared the total calories calculated using the Atwater factors.

Korean adults consume 17.7 mg of α-tocopherol equivalents (TE)/day which is larger than the adequate intake level of the KDRI at 12 mg TE/d [10]. However, the intake is actually inadequate because α-tocopherol only accounts for 22% of total vitamin E intake, while γ-, ɗ-, β-tocopherol accounts for 51.7, 12.6, 1.0%, respectively [10]. Almonds are an excellent source of α-tocopherol providing 7.4 mg per 28 g serving [20]. When almonds were consumed as part of Western diets, α-tocopherol intake was increased by 54–98% [18, 35]. Jambazian et al. [36] calculated that every 1% energy increase from almonds (~2.8 g) increases α-tocopherol status by 0.15 μmol/L. In our previous study, we found that 85 g/day of almonds added to the NCEP step 1 diet increased plasma α-tocopherol by 5.8% in patients with coronary heart disease [26]. The bioavailability of α-tocopherol is affected by habitual diets because of the requirement of fats to facilitate its bioavailability. We found that 56 g/day of almonds increased plasma α-tocopherol by 8.5% and decreased γ-tocopherol by 18.1%, magnitudes of the change consistent with the results of studies conducted in Americans and Chinese [15, 36]. Since 23% of Korean adults are vitamin E deficient based on the plasma α-tocopherol concentration [10], our results support the notion that almonds can be an effective food to prevent vitamin E deficiency and inadequacy in Koreans. The improvement in α-tocopherol status after almond consumption appears more marked when expressed the ratio α-tocopherol:TC, a more accurate values as tocopherols are transported via lipoproteins and their circulating concentrations are associated with TC [36].

A recent meta-analysis of 18 randomized controlled trials showed that almonds improved TC, LDL-C, and TG statuses but had no effect on HDL-C [21]. The favorable effects of almonds on serum lipid profiles proceed in a dose-responsive manner, particularly among individuals with hyperlipidemia [37–39], with a 1% reduction in LDL-C associated with each 7 g of intake [33, 39, 40]. The hypocholesterolemic effect of almonds appears to extend to patients taking statins [41]. Similarly, Lee et al. [42] found that daily consumption of 30 g of mixed nuts for 6 weeks decreased TC by 4% in Korean women with metabolic syndrome. Further, the addition of nuts to Korean diets may help control the lipid profile among those at increased risk for non-alcoholic fatty liver disease [43]. Consistent with these data, we found that almonds consumed as a snack were beneficial to lipid profile in Koreans as compared to cookies. Korean Guidelines for the Management of Dyslipidemia suggest nuts as an appropriate fat source [3]. In addition, the guidelines recommend limited intakes of biscuit, cookie, and cake, which rank 13th~14th of the main sources of fat intake in Korean diet [2]. These confectionary foods are major processed food sources of total sugar intake in Koreans [44, 45]; however, frequent consumption of sweet snacks is not consistent with dietary patterns for heart health [8, 9]. In contrast, the nutritional profile and impact on lipid profiles suggest almonds as a better snack. Nevertheless, among the main sources of fat in Korean diet, almonds rank 26th with the estimated average almond intake 0.65 g/day, which provide 0.35 g/day of fat and contribute 0.15% daily energy intake [2]. Together with well consistent data on lipid profile in the literature, our study suggests that almonds shall be incorporated into Korean diet for heart health as Koreans consume approximately 0.65 g/day, which is much lower than the intake (1.5 oz or 42.5 g/day) recommended in the FDA qualified health claim for tree nuts [46].

The effects of almonds on oxidative stress and inflammation reported in the literature are mixed, likely due to variations in study design, subject inclusion criteria, etc. Jenkins et al. [33] found that 73 g/day of almonds decreased serum MDA of older subjects with dyslipidemia. However, Choudhury et al. [47] reported that 50 g/day of almonds did not affect plasma protein carbonyl or nitric oxide in adults with ≥2 CVD risk factors. The data in this study did not reveal any modification of three common biomarkers of oxidative stress, plasma protein carbonyls, MDA, and oxLDL. Similarly, Lee et al. [42] also obtained null results on biomarkers of oxidative stress in a study of mixed nuts in Koreans with metabolic syndrome. However, it is worthwhile noting that nuts, specifically almonds, peanuts and pine nuts are included in measures of dietary quality among Koreans which are inversely associated with measures of systemic lipid peroxidation [48].

With regard to biomarkers of inflammation, Rajaram et al. [49] reported that 68 g/day of almonds decreased serum hs-CRP and E-selectin, but did not affect serum IL-6 and fibrinogen. Liu et al. [17] found that almonds incorporated into an NCEP step 2 diet at 20% daily calories decreased IL-6 compared to controls, but did not alter ICAM-1 or VCAM-1 in Chinese patients with type 2 diabetes mellitus. We found the almond snack decreased IL-10 and tended to decrease serum ICAM-1, IL-1β and IL-6. Interestingly, a positive association between IL-10 and CVD risk has been reported among the elderly [50]. The absence of a statistically significant impact of almonds on the biomarkers of inflammation examined in this study may be due to their low concentrations at baseline which matched those of healthy, normal weight people [51, 52]. Nonetheless, a study of mixed nuts in Koreans with metabolic syndrome also found no effect on ICAM-1, VCAM-1, IL-6 or hs-CRP [42].

Daily supplementation of almonds in typical Korean diet for 4 weeks improves nutrient intakes, circulating vitamin E status, and lipid profiles in overweight and obese Koreans. However, there are several limitations in our study. Due to a relatively short intervention duration, the positive effects of chronic almond consumption on the outcome measures remain to be examined. Nutrient intake may not be accurately captured as values were calculated from self-reported dietary information. The sample size of this study may not be powered to detect the effect of almonds on TG, apo B, ICAM-1, IL-1β, and IL-6.

The impact of any food or nutrient on CVD risk must be evaluated against the background of ethnicity, genetics, diet, and lifestyle for the cohort studied. However, our almond intervention in a Korean population appears generally consistent with those found in other Asian as well as North American and European countries. This relationship may result from the positive nutrient attributes of almonds, i.e., their content of MUFA, PUFA, fiber, and vitamin E, and their combined association with heart health.

## Electronic supplementary material

Below is the link to the electronic supplementary material.

Supplementary material 1 (DOCX 12 kb)

## Abbreviations

Apo A: Apolipoprotein A
Apo B: Apolipoprotein B
BMI: Body mass index
CVD: Cardiovascular disease
HDL-C: High-density lipoprotein cholesterol
hs-CRP: High-sensitive C-reactive protein
ICAM: Intercellular adhesion molecule
IL: Interleukin
KNHANES: Korea National Health and Nutrition Examination Survey
LDL-C: Low-density lipoprotein cholesterol
MDA: Malondialdehyde
MUFA: Monounsaturated fatty acids
oxLDL: Oxidized low-density lipoprotein cholesterol
PUFA: Polyunsaturated fatty acids
SFA: Saturated fatty acids
TC: Total cholesterol
TG: Triglyceride
TNF-α: Tumor necrosis factor-α
VCAM: Vascular cell adhesion molecule
