# Supplementary material for: The effect of almonds on vitamin E status and cardiovascular risk factors in Korean adults: a randomized clinical trial
Source: Eur J Nutr. 2017 Jul 10;57(6):2069–79. doi: 10.1007/s00394-017-1480-5 (PMC6105263; doi:10.1007/s00394-017-1480-5)
Supplement: Supplementary file 1 — Supplementary material 1 (DOCX 12 kb) [file 394_2017_1480_MOESM1_ESM.docx]

Supplemental Table 1. Neither almonds nor cookies affect body weight, body composition, and blood pressure of the subjects^a^

|  | Cookie | | Almond | |
| --- | --- | --- | --- | --- |
|  | Wk0 | Wk4 | Wk0 | Wk4 |
| Body weight (kg) | 66.2±8.7 | 66.4±8.9 | 66.3±8.7 | 66.3±8.8 |
| Waist circumference (cm) | 89.5±5.9 | 90.0±6.3 | 88.6±5.6 | 89.2±5.7 |
| BMI (kg/m^2^) | 25.4±2.1 | 25.5±2.0 | 25.4±2.0 | 25.4±2.1 |
| Body fat mass (kg) | 22.6±8.6 | 23.0±4.1 | 22.8±4.0 | 23.0±4.2 |
| SBP (mm Hg) | 122.1±16.1 | 120.3±16.1 | 118.7±12.4 | 116.8±13.5 |
| DBP (mm Hg) | 79.4±10.2 | 78.7±10.2 | 77.9±9.4 | 77.0±10.0 |

^a^Data are expressed as mean ± SD. BMI, body mass index; SBP, systolic blood pressure; DBP, diastolic blood pressure.
